# Physically Transient Memory on a Rapidly Dissoluble Paper for Security Application

**DOI:** 10.1038/srep38324

**Published:** 2016-12-05

**Authors:** Hagyoul Bae, Byung-Hyun Lee, Dongil Lee, Myeong-Lok Seol, Daewon Kim, Jin-Woo Han, Choong-Ki Kim, Seung-Bae Jeon, Daechul Ahn, Sang-Jae Park, Jun-Young Park, Yang-Kyu Choi

**Affiliations:** 1 School of Electrical Engineering, Korea Advanced Institute of Science and Technology, (KAIST) 291 Daehak-ro, Yuseong-gu, Daejeon, 34141, South Korea; 2 Center for Nanotechnology, NASA Ames Research Center, Moffett Field, California 94035, United States

## Abstract

We report the transient memory device by means of a water soluble SSG (solid sodium with glycerine) paper. This material has a hydroscopic property hence it can be soluble in water. In terms of physical security of memory devices, prompt abrogation of a memory device which stored a large number of data is crucial when it is stolen because all of things have identified information in the memory device. By utilizing the SSG paper as a substrate, we fabricated a disposable resistive random access memory (RRAM) which has good data retention of longer than 10^6^ seconds and cycling endurance of 300 cycles. This memory device is dissolved within 10 seconds thus it can never be recovered or replicated. By employing direct printing but not lithography technology to aim low cost and disposable applications, the memory capacity tends to be limited less than kilo-bits. However, unlike high memory capacity demand for consumer electronics, the proposed device is targeting for security applications. With this regards, the sub-kilobit memory capacity should find the applications such as one-time usable personal identification, authentication code storage, cryptography key, and smart delivery tag. This aspect is attractive for security and protection system against unauthorized accessibility.

As the technology of electrical devices advances, a cost-effective and flexible memory device has attracted a lot of attention in efforts to realize actual applications for general objects. Instead of conventional memory devices^1^,^2^ which are generally constructed on a Si substrate, in order to resolve aforementioned challenging issues, many research groups have attempted to construct memory devices on various substrates including paper, plastic, and fabric^3^,^4^,^5^,^6^ as an alternative. In particular, a paper is most widely used for a flexible substrate in field of electronics. Most of the paper has attractive merits in terms of low cost, flexibility, lightweight and eco-friendly material property. As the printing technique was introduced recently, paper-based electronics is ready to be realized. Up to now, many paper based applications have been developed, including non-volatile memory^3^, paper gated oxide transistors^7^,^8^,^9^,^10^, energy harvester^11^,^12^, and radio frequency identification (RFID) tags^13^ as well as resistors, capacitors, transistors, and diodes^14^,^15^,^16^,^17^. Among the electronic components implemented to a paper, RRAM is regarded as a promising non-volatile memory device because of its simple fabrication process, extreme low-cost, and high performance^18^. Accordingly, a highly adaptable flexible RRAM has attracted a great deal of attention as one of key elements to flexible electronics or living objects. On the other hand, such memory devices should be protected to secure sensitive data from exposure to public or being stolen beyond robust functionality for data storage. Thus, immediate protection of the stored data is essentially required against a security problem caused by unauthorized access. In this respect, a memory device should be disposable easily and quickly. In the worst case, the permanent and physical destruction of a memory device is needed for the highest level of system security.

In this study, a water soluble disposable memory device with a flexible characteristic and low-cost process, is demonstrated for security applications. Disposable electronic devices, such as a water soluble device^19^,^20^,^21^,^22^,^23^,^24^ and physically transient electronics^25^,^26^,^27^ have previously been considered for biocompatible electronics and biological therapies^22^,^28^. Notably, by Rogers group, silicon-based transient electronics is investigated lately^19^,^23^. Such physically transient electronics shows the possibilities of soft electronics which can be expand opportunities for biomedical devices such as implantable medical diagnosis. In addition, in the near future, the water soluble feature of memory devices can also be utilized for security enhancement and play a significant role in the field of the transient electronics. For this, the resistive random access memory (RRAM) on a paper system was employed. The RRAM is a promising candidate of a flexible memory component due to its high packing density and simple metal-insulator-metal (MIM) structure^29^,^30^,^31^,^32^,^33^. It was implemented onto a dissoluble paper made of solid sodium with glycerine (SSG) hence it is dissolved within approximately 10 seconds. The role of the SSG based paper substrates is to provide a mechanical support as a substrate and to facilitate the dissolution of the device into the water. Therefore, the SSG based paper itself does not involve the electrical behavior of the memory. As a control group, we fabricated the RRAM on a commercial sticker-type paper. This type of paper is mostly composed of cellulose, while the paper substrate of our proposed memory consists of sodium and glycerine. In the SSG substrate, the hydrophilic group (-COONa) reacts with water molecule and dissolves rapidly in the water. In order to realize a rapidly disposable memory device, the inkjet printing technique was used. The inkjet printing is favorable for well-designed metal patterns at room temperature without any damage of a substrate. By utilizing the SSG substrate, it is expected that a disposable memory device to store security data can be implemented to a universal serial bus (USB) card and discarded after reading.

## Results and Discussion

**Figure 1:**
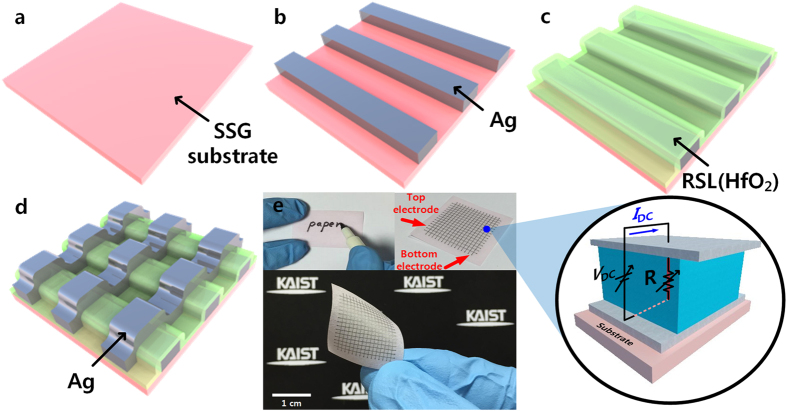
(**a**) to (**d**) show schematics of the proposed disposable memory device with rapidly dissoluble substrate (**a**) ultrathin substrate including inherent hydroscopic property based on solid sodium with glycerine (SSG). (**b**) Deposition of bottom electrode (silver) by inkjet printing technique (**c**) Deposition of HfO_2_ (10 nm) as resistive switching layer (RSL) by atomic layer deposition (ALD) process. (**d**) Deposition of top electrode (silver) by inkjet printing technique. (**e**) A photograph of the dissoluble and flexible memory device and enlarged view of unit cell. The inset shows the image of the fabricated memory device in an unfold state.

Fig. 1(a) to (d) show the schematics of the device fabrication procedure, and the device structure of the proposed RRAM. This RRAM has a simple capacitor-like metal-insulator-metal (MIM) structure which consists of an HfO_2_ restive switching layer (RSL) between top electrode (TE) and bottom electrode (BE). Figure 1(e) illustrates real images of the fabricated RRAM on the SSG substrate.

**Figure 2:**
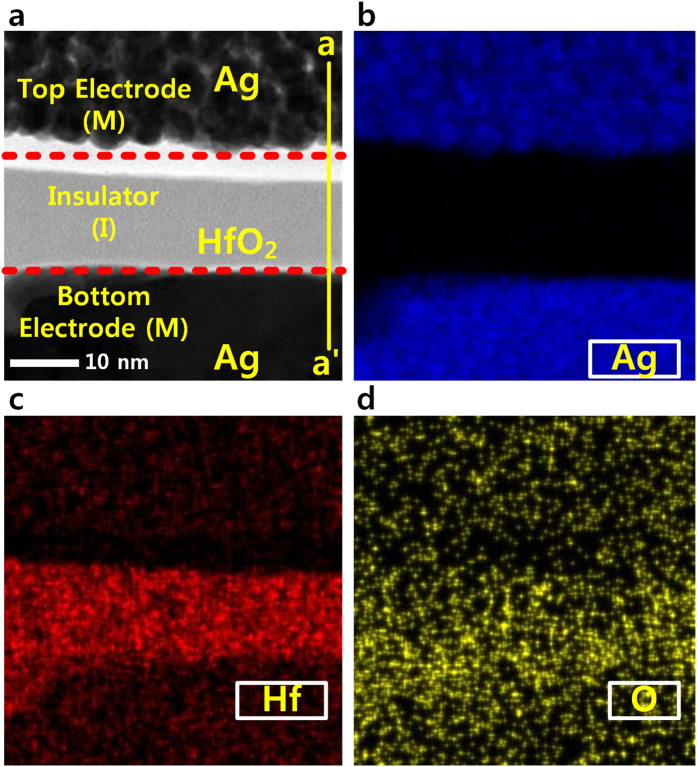
TEM image of the fabricated RRAM (**a**) Cross-sectional TEM image of the metal-insulator-metal (MIM) structure along the a-a’ direction. (**b**,**c** and **d**) are EDS mapping images captured for analyzing the Ag component of top and bottom electrodes as well as the Hf and O components of the insulator, respectively.

The transmission electron microscopy (TEM) images of the fabricated RRAM are illustrated in Fig. 2(a). Figure 2(a) presents a cross-sectional view of the RRAM with ‘metal-insulator-metal’ structure. The HfO_2_ as a RSL is formed with about 10 nm thickness. Figure 2(b) to (d) show elemental mapping images obtained by energy dispersive spectroscopy (EDS), which investigates the components (Ag, Hf, O) in each layer.

**Figure 3:**
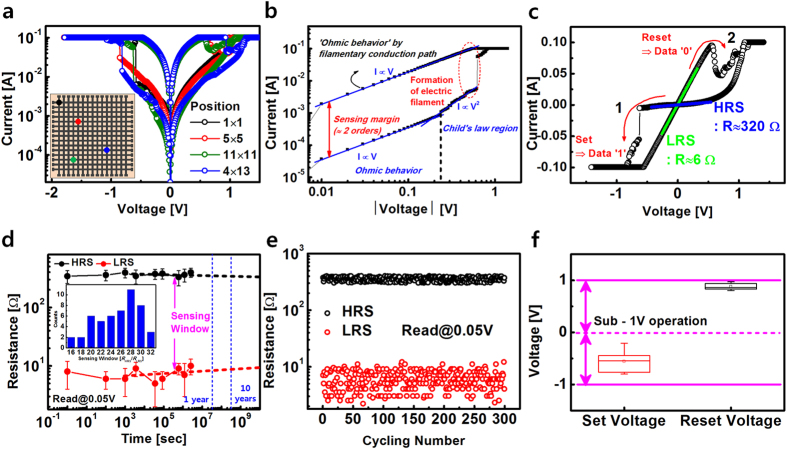
Experimental analysis of the fabricated RRAM device on SSG substrate. (**a**) The normal current-voltage characteristics for memory operation with bipolar switching mode at various position on array structure. The inset shows an image of the fabricated 15 × 15 matrix memory device on a SSG substrate. (**b**) Current-voltage (log I-log V) relation curve in ‘SET’ process. The inset shows (log I)-V characteristic. (**c**) Switching mechanism for the fabricated memory device. While ‘1’ process leads to the LRS which has low resistance value of sub-10 Ω, ‘2’ process give rise to the HRS which has a high resistance value of about hundreds of Ohm. Resistance change (HRS: 320 Ω → LRS: 6 Ω) through ‘SET’ process by formation of electric conducting filament employing oxygen vacancy as electrical channel of electrons. (**d**) The retention behavior of the HRS and LRS as function of time. (**e**) The variation of the HRS and LRS as a function of the retention time and switching cycles **(f**) SET and RESET voltage distributions of the RRAM described by a box-whisker plot obtained from I-V curves of 50 unit cells.

Figure 3(a) to (f) show the electrical characteristics of the proposed memory device. Figure 3(a) shows the representative current-voltage (*I*-*V*) curves measured from different devices located at various positions in the array. Figure 3(a) confirms that the fabricated RRAM has nonvolatile memory characteristics. This RRAM exhibits bipolar resistive switching behavior with very low operating voltage (<1 V) and wide memory window (approximately 2 orders). Also, Fig. 3(a) shows the non-polar switching characteristics regardless of bias polarity (Supporting Information)^34^. The inset of Fig. 3(a) shows a 15 × 15 matrix RRAM fabricated on the SSG substrate with a size of 1.5 × 1.5 cm^2^. By measuring representative *I*-*V* characteristics in the array cells, it was verified that the fabricated RRAM has high uniformity. However, the fabricated RRAM shows a high level of current arisen from a large cell size, which is 100 μm × 100 μm and a simple MIM structure^35^. Therefore, it is required to suppress the current level by further miniaturization and structural optimization. It is noteworthy that the demonstrated rapid disposability of memory built on the SSG paper can be applicable to a high level of security application.

Figure 3(b) shows the resistive switching mechanism which can be explained using the filament model^36^. Oxygen vacancies drift towards the top electrode under an electric field produced by an applied negative bias^37^. When oxygen vacancies are connected between the top electrode and the bottom electrode, these electric filaments generate a low resistance state (LRS)^38^. In turn, a high resistance state (HRS) is produced by rupturing the filaments with application of a positive bias, which is opposed to the bias to change from LRS to HRS.

In order to investigate the conduction mechanisms of the LRS and HRS, the *I*-*V* characteristics were re-plotted on double-logarithmic scales as shown in Fig. 3(b). The *I*-*V* curve follows a linear ohmic conduction. This indicates that the resistive switching phenomenon in both the LRS and HRS can be explained by the formation and rupture of electric filaments in the RSL^39^,^40^,^41^.

Figure 3(c) shows the change of resistance value produced by the formation (SET process) and rupture (RESET process) of the electric filaments. In the ‘1’ process, when the TE voltage is shifted from zero to a high voltage in the negative direction, this behavior leads to the LRS which represents the “data 1” in memory. In contrast to this, when the TE voltage is swept from zero to a high voltage in the positive direction, this behavior give rise to the HRS, which indicates the “data 0” in memory.

The resistance change from HRS (320 Ω) to LRS (6 Ω) was made through the ‘SET’ process by the formation of electric filaments. In that process, connected oxygen vacancies serve as an electrical channel for electrons.

Figure 3(d) and (e) show the data retention and endurance properties of the RRAM. Current reading was done under a read voltage of 0.05 V. There was no perceivable degradation in the data retention up to 2 × 10^6^ seconds (23 days), which by extrapolation is expected to prolong to 10 years. The dashed line is the expectancy of data storage capability while maintaining the programmed state (HRS or LRS). The sensing window (*R*_HRS_/*R*_LRS_) is distributed over a limited range (16~32). These experimental results were averaged by repeated measurements (50 times). Furthermore, Fig. 3(e) shows that the RRAM can be operated for over 300 program/erase cycles without degradation. It is noteworthy that the fabricated RRAM can operates without the electro-forming process due to the thin HfO_2_ layer^42^,^43^,^44^. Figure 3(f) shows the fluctuation of the SET and RESET voltage, which are defined at the voltage to show that current is abruptly increased at the measured *I*-*V* curves. The distribution of the SET voltage is slightly broader than that of the RESET voltage. Furthermore, the AC pulse response, non-polar *I*-*V* characteristics, and temperature instability of the fabricated RRAM are described in Supporting Information.

**Figure 4:**
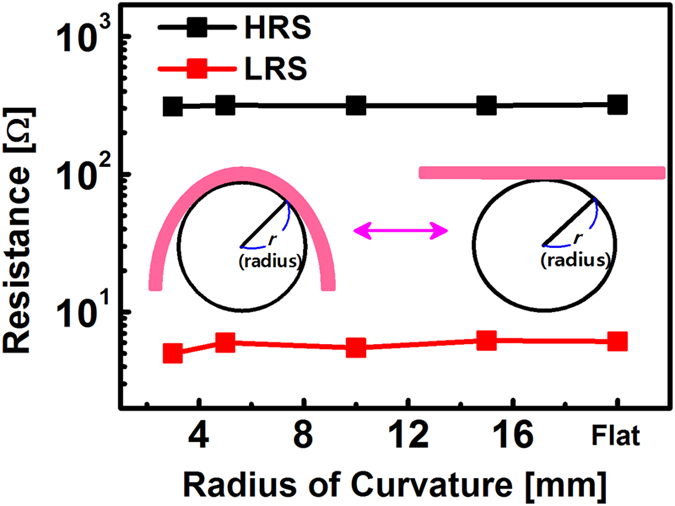
The variation of the HRS and LRS as a function of the bending radius (3 mm to flat state (∞)) of the fabricated RRAM on the SSG substrate with inherent flexibility.

The fabricated RRAM also shows good device performances even under bending conditions. Especially, the fabricated RRAM on the SSG substrate maintains a bended state under external bending force due to intrinsically negligible restoring force, which was usually found in a conventional paper. Experimental results regarding durability against the iterative bending are shown in Fig. 4 and Supporting Information 7. In order to perform the bending test, the fabricated RRAM on the SSG substrate was loaded onto a semicircular holder then *I*-*V* characteristics were measured. The fabricated RRAM on the SSG substrate shows stable memory operations with a bending radius of 3 mm. Figure 4 shows the inherent flexibility of the SSG substrate with negligible elasticity. This feature shows that the fabricated RRAM on the SSG substrate has the potential to attach to an irregular structure. As shown in the Fig. 4, there are no perceivable changes of HRS and LRS according to the bending curvature. The measured *I*-*V* curves with various bending radius are shown in Supporting Information. In light of these experimental results, the fabricated RRAM on the SSG substrate can be served as flexible memory for any objects with irregular surface.

**Figure 5:**
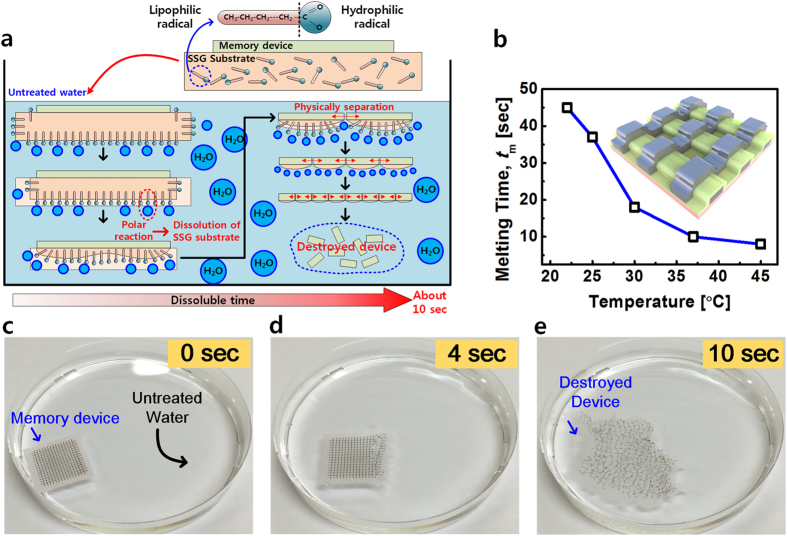
Decomposition mechanism of the fabricated memory device and photographs for experiment of the fabricated memory on rapidly dissoluble substrate in untreated water at various temperature step. (**a**) Schematics of the melting sequences of the fabricated memory device on the SSG substrate in untreated water. (**b**) The melting time vs. water temperature plot for submerged device on dissoluble substrate. The inset shows a schematic image of the fabricated memory device. (**c**) to (**e**) show optical photographs for dissolution sequence of underwater experiment of the fabricated memory on rapidly melting substrate in water for about 10 s. (**c**) Image of a device just soaked in water. (**d**) The decomposed device in water after 4 s. (**e**) Image of thoroughly destroyed device by water after about 10 s.

Figure 5 demonstrates the water soluble characteristics of the proposed device on the SSG substrate. Figure 5(a) shows schematics of decomposition mechanism. By chemical reaction of the carboxyl group of the SSG substrate and water molecule, the SSG substrate starts to melt in untreated water. After a few seconds, some parts of the SSG substrate are rapidly divided from microscopic parts because the SSG substrate has excellent water solubility. By continuous decomposition of the SSG substrate, the fabricated device on the SSG substrate can be physically destroyed. Figure 5(b) shows a plot of melting time (*t*_*m*_) versus water temperature measured for the submerged device. As the water temperature increases, the SSG substrate melts more quickly, although *t*_*m*_ starts to saturate over 35 °C. Figure 5(c) to (e) show optical photographs which display the sequence of the dissolution process after immersion in tap water for 10 seconds. Within several seconds of being dipped in tap water, the RRAM device begins to be divided into a few pieces and is completely and physically melted within approximately 10 seconds. After that, recovery of the programmed data in the memory device is impossible owing to the physical destruction. Figure 5(c) shows an image of the memory device just being placed in the tap water, Fig. 5(d) exhibits it after 4 s, and Fig. 5(e) represents it after 10 seconds, respectively. Once the memory device has been melted, such devices cannot be returned to an original form. Therefore, unauthorized users cannot have access to any stored data. In comparison with previous studies on the physical dissolubility of biodegradable and water-soluble materials^11^,^14^, the physical disposable time of the dissoluble paper is dramatically reduced, and its cost was extremely low in this work because it was already commercialized and under mass production.

In summary, a rapidly disposable and flexible non-volatile memory device was demonstrated using the dissoluble paper made of solid sodium with glycerine (SSG). The proposed RRAM device showed distinctive characteristics, i.e., quick disposal speed compared to other water soluble polymer devices, and low-cost. Moreover, the fabricated memory device showed improved memory performances in terms of a large memory window of approximately 2 orders of magnitude, low operating voltage of below 1 V, and device stability confirmed by retention time of 1 month and cyclic endurance of up to 300 cycles. In this paper, we demonstrate that the security of important data can be guaranteed based on the disposability of the fabricated memory device on SSG substrate.

Additionally, because it can be fabricated using very familiar device fabrication processes including inkjet printing technique and the ALD process, the proposed memory device on SSG substrate has two definite advantages, cost and simple structure. Furthermore, the disposable memory device uses a previously qualified RRAM with the simplest structure, and also had good memory operation based on reliable and reproducible resistive switching behavior, including bending cycle, retention time, and repetitive endurance cycles. From these results, therefore, the RRAM on the SSG substrate can potentially serve as a memory array of fundamental building blocks for integration into future rapidly disposable electronics.

## Methods

### Fabrication of the device

As shown in Fig. 1, a SSG paper substrate composed of sodium, glycerine, and glutamate is commercially available and it was prepared with a size of 1.5 × 1.5 cm^2^. The SSG substrate includes inherent hydroscopic and flexible properties. The bottom electrode (BE) with a thickness of 100 μm was directly patterned on the dissoluble paper by inkjet printing without any additive surface treatment. For this, ink composed of silver (Ag) nanoparticles was used. Afterwards, HfO_2_ of a thickness of 10 nm was deposited by the atomic layer deposition (ALD) as the RSL. Using this monolayer deposition, excellent and high-quality thin-film fabrication is possible. In order to form the HfO_2_ layer stably without damage to the SSG substrate, the process temperature was optimized at approximately 250 °C as described in Supplementary Information. Thereafter another silver layer was printed onto the HfO_2_ layer as the top electrode (TE) via the same direct inkjet printing technique.

### Experimental equipment

All electrical measurements were carried out in an ambient air environment without any device encapsulation. The electrical I-V characteristics were measured by using an HP4156 semiconductor parameter analyzer. The AC pulse-induced electrical data are measured by employing Tektronix TDS2024B (pulse generator) and agilent 81110 A (digital oscilloscope). An AFM (model XE100) analysis was also conducted to assess the surface roughness of the Ag-coated SSG substrate and pure SSG substrate. In order to verify the reliability and stability of the fabricated disposable memory in moisture environment, the humidity experiment was performed by using a thermos-hygrostat (model TE-ME). The relative humidity (RH) is a ratio between the amount of water vapor in the air and the maximum amount of water vapor required for saturation at a specific temperature. In the TEMI 300 (TE-ME) chamber, the humidity experiment was conducted under the various relative humidity values from 20% to 95% at a room temperature (22 °C) during 1 hour. In terms of the second supplementary video in Supplementary Information, the pure blood is extracted from the carcass (dead pig) in slaughterhouse. Thus, it does not require approval and has already been conformed.

### Dissolution experiment

Complete decomposition time of the fabricated memory device on the SSG substrate was defined as the moment that the SSG substrate completely dissolves and each memory site linked by the top and bottom electrode is physically disconnected in the water. All the experiments were repeated five times and experimental data were averaged.

## Additional Information

**How to cite this article**: Bae, H. *et al*. Physically Transient Memory on a Rapidly Dissoluble Paper for Security Application. *Sci. Rep.* **6**, 38324; doi: 10.1038/srep38324 (2016).

**Publisher's note:** Springer Nature remains neutral with regard to jurisdictional claims in published maps and institutional affiliations.

## Supplementary Material

Supplementary Video 1

Supplementary Video 2

Supplementary Information
