# Supplementary material for: Physically Transient Memory on a Rapidly Dissoluble Paper for Security Application
Source: Sci Rep. 2016 Dec 5;6:38324. doi: 10.1038/srep38324 (PMC5137035; doi:10.1038/srep38324)
Supplement: Supplementary Information [file srep38324-s3.doc]

**Supplementary Information**

**Physically Transient Memory on a Rapidly Dissoluble Paper for Security Application**

*Hagyoul Bae^†1^,Byung-Hyun Lee^†1^, Dongil Lee^1^, Myeong-Lok Seol^2^, Daewon Kim^1^, Jin-Woo Han^2^, Choong-Ki Kim^1^, Seung-Bae Jeon^1^, Daechul Ahn^1^, Sang-Jae Park^1^, Jun-Young Park^1^, and Yang-Kyu Choi*^1^*

^1^School of Electrical Engineering, Korea Advanced Institute of Science and Technology, (KAIST) 291 Daehak-ro, Yuseong-gu, Daejeon, 34141, South Korea

^2^Center for Nanotechnology, NASA Ames Research Center, Moffett Field, California 94035, United States

† These authors equally contributed to this work

*Authors to whom correspondence should be addressed.

Email address: [ykchoi@ee.kaist.ac.kr](mailto:ykchoi@ee.kaist.ac.kr)

**1: Procedure of Melting Device (Video)**

Two videos are **uploaded to the online submission system** as below**.**

**Video 1 :** A melting procedure of the fabricated memory device in untreated water.

**Video 2 :** A melting procedure of the fabricated memory device in pure blood.

**2: Surface Morphology Characterizations Using Atomic Force Microscopy (AFM)**

**Fig S2** shows atomic force microscopy (AFM) images of the silver layer on the HfO_2_ layer and the silver-coated SSG substrate. As shown in **Fig S2a**, morphology of the silver layer on HfO_2_ layer is uniform because the subsequently deposited HfO_2_ layer with the aid of the ALD has a thickness of less than a few tenths of nanometer. **Fig S2b** shows the surface roughness of the silver-coated SSG substrate by screen printing technique. After the silver coating process, the RMS value of silver coated SSG substrate is reduced. Silver coating is an important step to make a conformal and uniform profile of the RSL, which is made of HfO_2_.

**
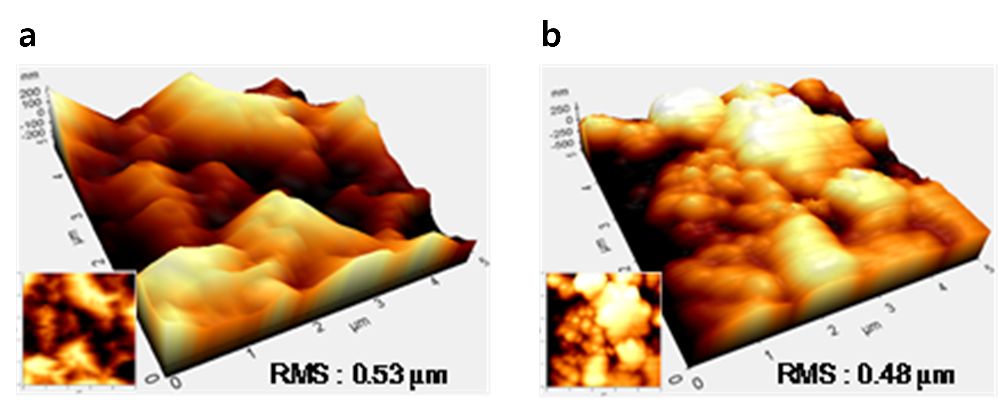
**

**Fig S2**

**3: Inkjet Printing Technique**

**Conductive silver ink and inkjet printing process**: The silver layer as the TE and BE was deposited by inkjet printing technique. Conductive silver ink (TEC-IJ-060) was purchased from the company (InkTec Co., Ltd.). The printed silver is not formulated as a form of a particle hence it is stable even under normal temperature. In order to enhance electrical conductivity of the metal (silver) electrode, sintering process is required before the printing (approximately 30 min at 100~150 ℃). At the room temperature, viscosity and density of the inkjet ink are 5 ~ 15 cps and 1.07 g/cm^3^, respectively. In order to form matrix electrode, we use an ‘Inkjet Printer (UJ-200 MF)’. The nozzle diameter was about 50 μm. The maximum jetting frequency was set to 3 kHz. A printed line-shaped pattern is obtained by depositing drops next to each other. For the fabricated memory device, a distance between each line pattern is 1 mm. A width and a thickness of the Ag electrode are 100 μm and 1 μm, respectively.

**4: Conductivity Characteristics of Silver Electrodes**

Silver was deposited by the inkjet printing technique. The TE and BE can be damaged by relatively thick probe tip so we normally use a straight-type probe tip because it has small contact edge. In addition, **Fig S4** exhibits the Ohmic relation with resistance of 5 Ω when we measured metal (Ag) conductivity by use of the abovementioned probe tips. It is confirmed that the Ag electrodes in the top and the bottom were well deposited by inkjet printing. It is noteworthy that the voltage application without voltage drop in the top and bottom metal line is crucial for the stable formation of electric filament in RSL. In order to clarify the inkjet printing process, we added a measured data of the conductivity of Ag electrode as follow:

**Fig S4**

**5: Humidity Experiment**

**Fig S5** shows the result of humidity experiment of the SSG substrate. As shown in the **Fig S5a**, the humidity experiment with respect to relative humidity (RH) was performed by using a thermos-hygrostat (model TEMI 300). Even though the RH increases, the SSG substrate is not nearly decomposed. This feature is very attractive to warrant the reliability and stability even when the proposed disposable memory is exposed to air. As shown in **Fig S5c,** unlike **Fig S1**, the SSG substrate is insensitive to the RH because amount of the water molecule in the air is much smaller compared with that in water. A range of humidity for this experiment is from 20% to 95%. Conclusively, the SSG substrate is not melted by gaseous moisture but by liquid moisture or water. Even under 95% relative humidity, RRAM characteristics are negligibly changed as described in **Fig S5d**. These experiments assure that the proposed device is easily and intentionally destroyed for security applications in the water while it is not damaged in the air.


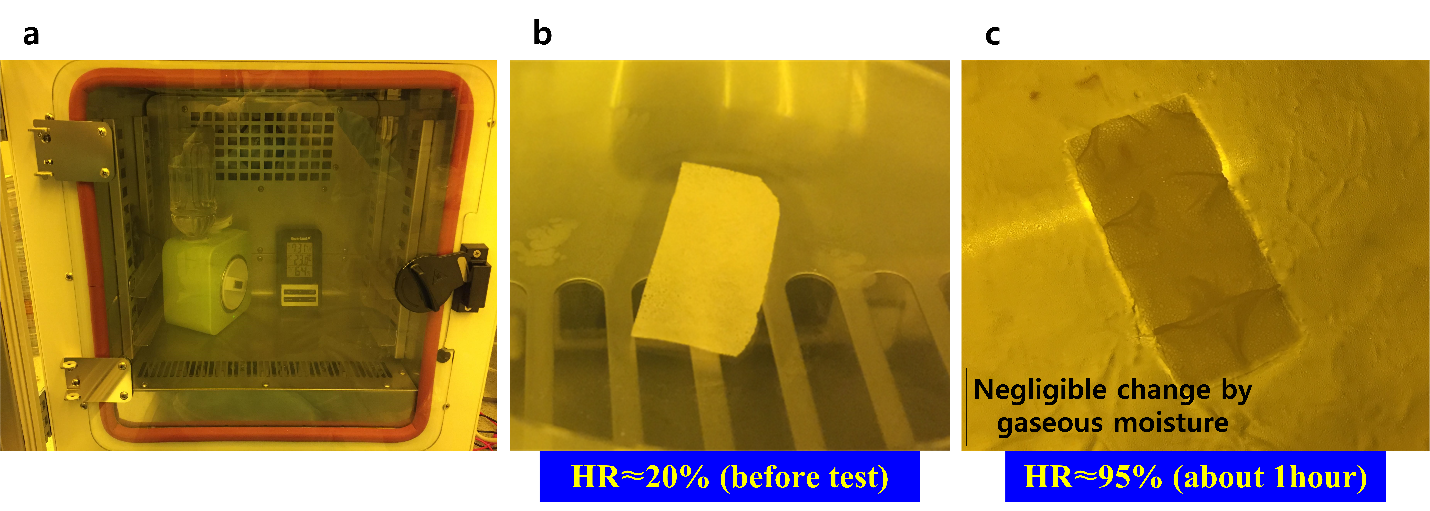


**Fig S5**

**6: Non-Polar Operation**

**Fig S6** shows the non-polar switching characteristics because both top electrode (TE) and bottom electrode (BE) consist of same metal material (silver).

**
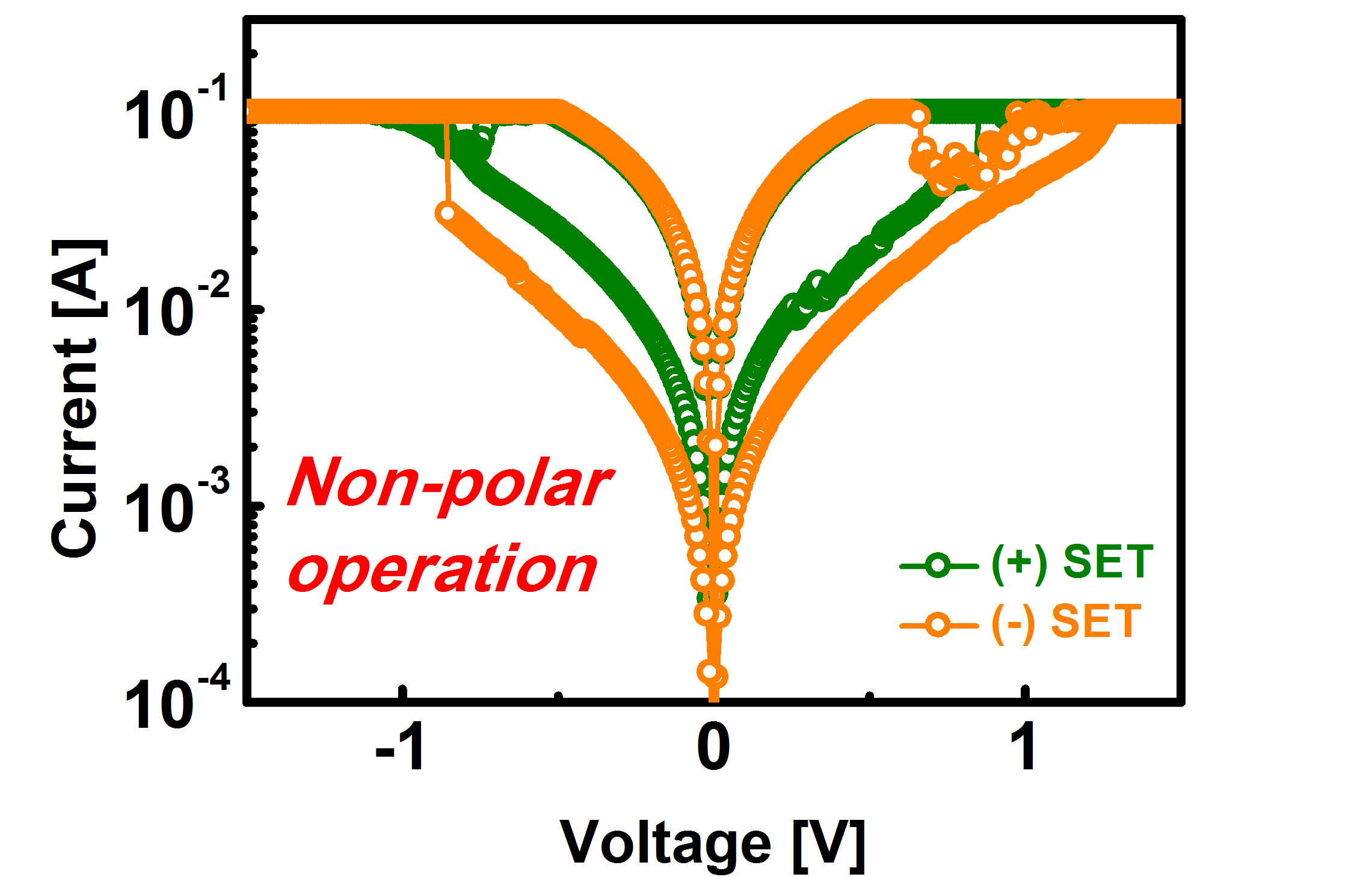
**

**Fig S6**

**7: Bending Test**

**Fig S7** shows the *I*-*V* characteristics with various the bending radius (3 mm to flat state) as described in manuscript. The fabricated device shows an excellent bendability.

**
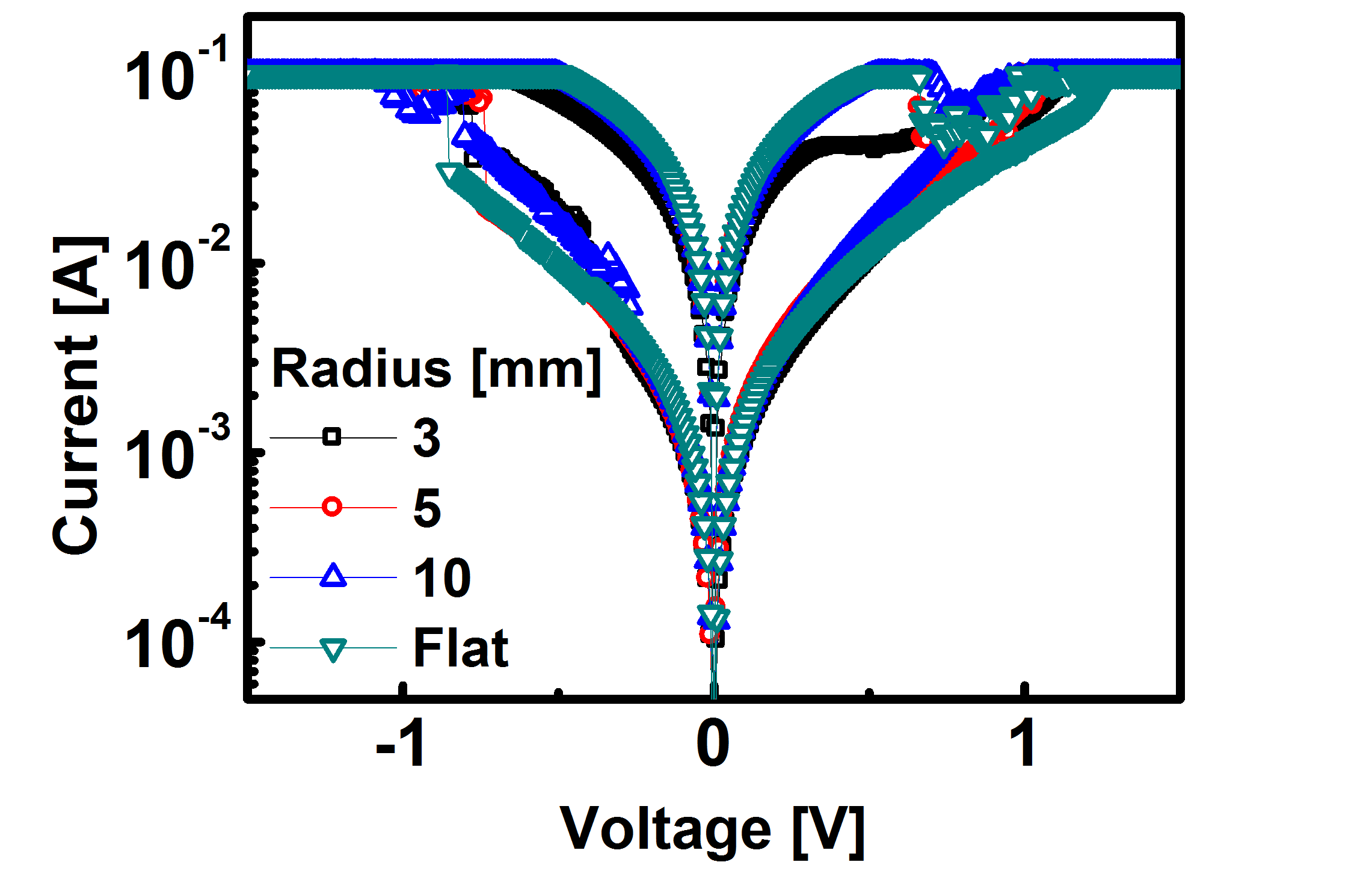
**

**Fig S7**

**8: AC Pulse-Induced Switching Characteristics**

In terms of the AC pulse response, the switching endurance of the fabricated RRAM is demonstrated in **Fig S8**. (pulse width: 50 ns for set pulse / 50 ns reset pulse). After AC endurance test, the change of the resistance values are negligible in the HRS/LRS as below:


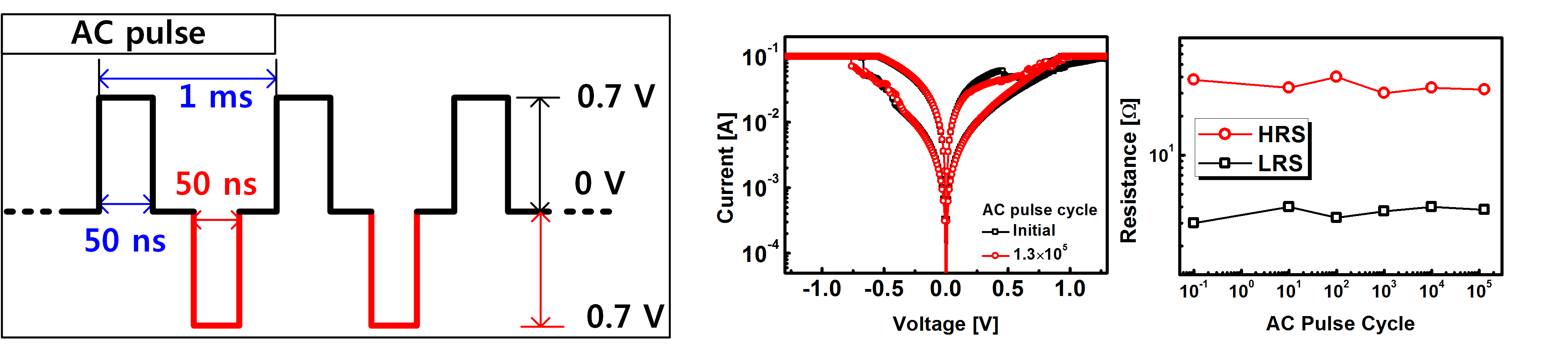


**Fig S8**

**9: Temperature-Dependency**

In order to confirm the temperature stability, we measured the fabricated RRAM on hot-chuck measurement system at room temperature and 85℃ as shown in **Fig S9**. As the device temperature increases, the leakage current in the HRS increases owing to increased trap-assisted tunneling. Also, the set and reset voltages decrease because of lower potential barrier.

**
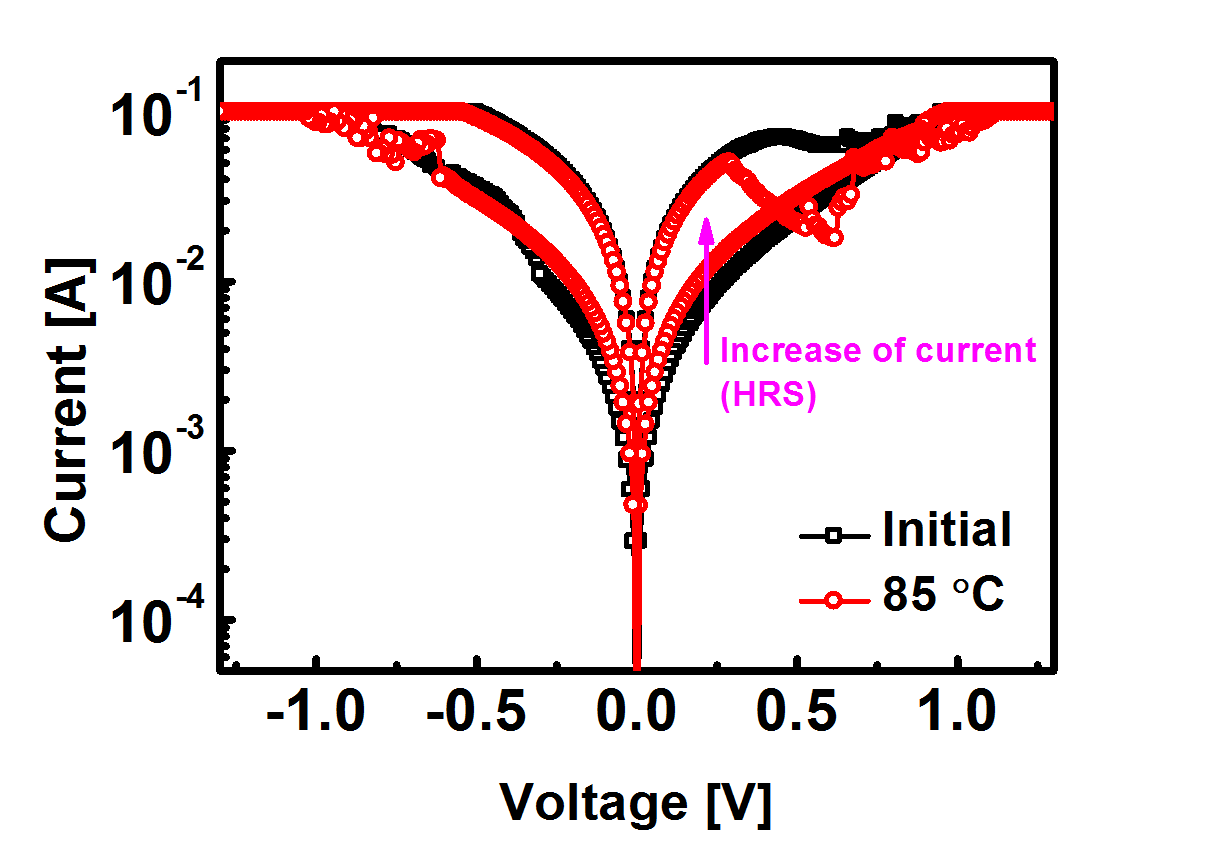
**

**Fig S9**

**10: XRD and XPS Analysis**

We prepared two HfO_2_ film sample for crystalline analysis. As shown in XRD plot of **Fig S10**, crystalline HfO_2_ film is formed at 250 °C showing temperature dependency while amorphous HfO_2_ film is formed at 100 °C. Since, in our experiments, we used the HfO_2_ film with 250 °C, HfO_2_ film is crystalline. In addition, for further element analysis, we performed XPS spectroscopy. As a control sample, we prepared bare SSG substrate. And we prepared HfO_2_ (10 nm) deposited onto SSG substrate as an experimental sample. In Hf 4f of XPS spectrum below, HfO_2_ film (10 nm) was successfully deposited onto SSG substrate.


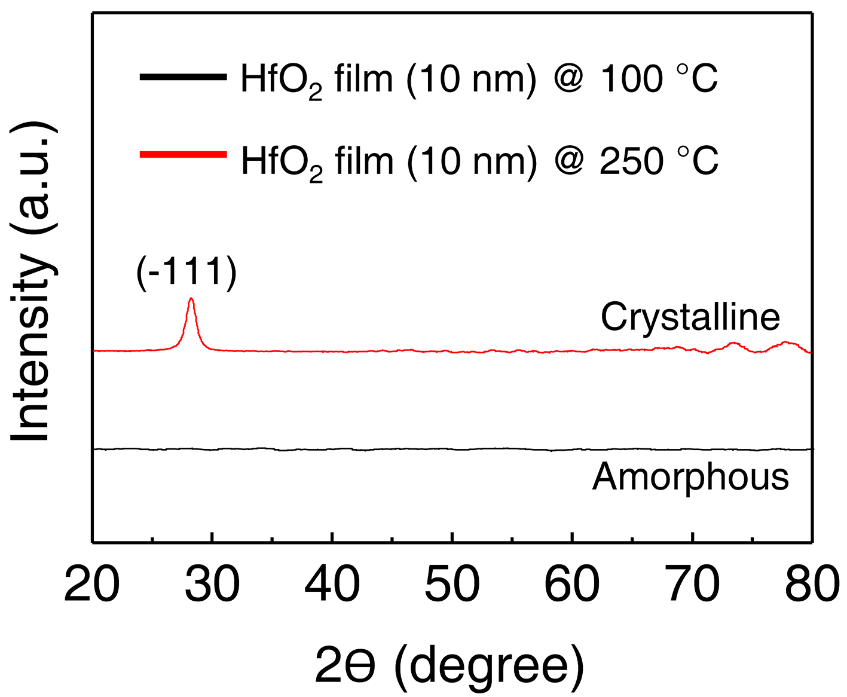

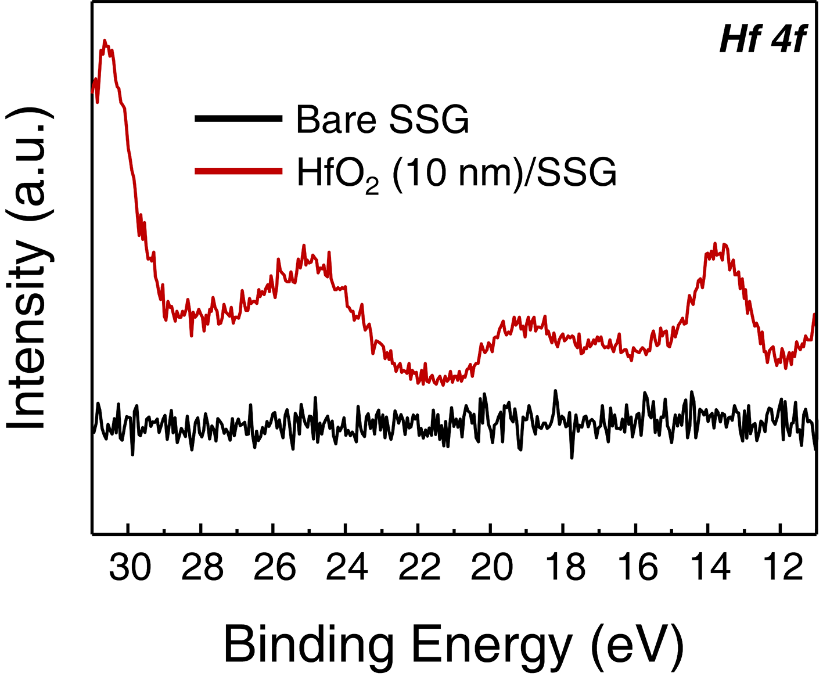


**Fig S10**

**11: Reproducibility of Memory Operation**

We demonstrated the reproducibility of the measurements including error bar as shown in **Fig S11**.

**Fig S11**

**SUPPORTING INFOMRATION REFERENCE**

1. Kim, D.-H. et al. Silicon Electronics on Silk as a Path to Bioresorbable, Implantable Devices. *Appl. Phys. Lett*, **95**, 133701-1‒133701-3 (2009).
2. Kim, S. et al. Tuning Resistive Switching Characteristics of Tantalum Oxide Memristors through Si Doping. *ACS Nano* **10**, 10262‒10269 (2014).
3. Hwang, S.-W. et al. A Physically Transient Form of Silicon Electronics. *Science* **337**, 1640‒1644 (2012).
4. Ji, Y. et al. Stable Switching Characteristics of Organic Nonvolatile Memory on a Bent Flexible Substrate. *Adv. Mater.* **22**, 3071‒3075 (2010).
5. Sirringhaus, H. et al. High-Resolution Inkjet Printing of All-Polymer Transistor Circuits. *Science*, **290**, 2123‒2126 (2000).
6. Seifert, T. et al. Additive Manufacturing Technologies Compared: Morphology of Deposits of Silver Ink Using Inkjet and Aerosol Jet Printing. *Indus. & Eng. Chem. Research,* **54**, 769‒779 (2015).
7. Bersuker, G. et al. Metal Oxide RRAM Switching Mechanism Based on Conductive Filament Microscopic Properties. *IEEE Tech. Dig.* 456‒459 (2010).
8. Cagli, C. et al. Experimental and Theoretical Study of Electrode Effects in HfO_2_ based RRAM. *IEDM Tech. Dig.* 658‒661 (2011).
9. Fang, Z. et al. Temperature Instability of Resistive Switching on HfO_x_-Based RRAM Devices. *IEEE Electron Device Lett.* **31**, 476‒478 (2010).
